# Blood Krebs von den Lungen-6 levels predict treatment response to antifibrotic therapy in patients with idiopathic pulmonary fibrosis

**DOI:** 10.1186/s12931-022-02273-6

**Published:** 2022-12-09

**Authors:** Myeong Geun Choi, Sun Mi Choi, Jae Ha Lee, Joong-Yub Kim, Jin Woo Song

**Affiliations:** 1 Department of Pulmonary and Critical Care Medicine, Asan Medical Center, University of Ulsan College of Medicine, 88, Olympic-ro 43-gil, Songpa-gu, Seoul, 05505 South Korea; 2 Division of Pulmonary and Critical Care Medicine, Department of Internal Medicine, Mokdong Hospital, College of Medicine, Ewha Womans University, Seoul, South Korea; 3 Division of Pulmonary and Critical Care Medicine, Department of Internal Medicine, Seoul National University Hospital, Seoul National University College of Medicine, Seoul, South Korea; 4 Division of Pulmonary and Critical Care Medicine, Department of Internal Medicine, Inje University Haeundae Paik Hospital, Inje University College of Medicine, Busan, South Korea

**Keywords:** Krebs von den Lungen-6, Idiopathic pulmonary fibrosis, Antifibrotic treatment

## Abstract

**Background:**

Antifibrotic therapy can slow disease progression (DP) in patients with idiopathic pulmonary fibrosis (IPF). However, the prognostic biomarkers for DP in patients with IPF receiving antifibrotic therapy have not been identified. Therefore, we aimed to evaluate the prognostic efficacy of serum Krebs von den Lungen-6 (KL-6) for DP in patients with IPF receiving antifibrotic therapy.

**Methods:**

The clinical data of 188 patients with IPF who initiated antifibrotic therapy at three tertiary hospitals was retrospectively analyzed. DP was defined as a relative decline in forced vital capacity (FVC) ≥ 10%, diffusing capacity for carbon monoxide ≥ 15%, acute exacerbation, or deaths during 6 months after antifibrotic therapy.

**Results:**

The mean age of patients was 68.9 years, 77.7% were male, and DP occurred in 43 patients (22.9%) during follow-up (median, 7.6 months; interquartile range, 6.2–9.8 months). There was no difference in baseline KL-6 levels between the DP and no-DP groups; however, among patients with high baseline KL-6 levels (≥ 500 U/mL), changes in KL-6 levels over 1 month were higher in the DP group than those in the non-DP group, and higher relative changes in KL-6 over 1 month were independently associated with DP (odds ratio, 1.043; 95% confidence interval 1.005–1.084) in the multivariable logistic analysis adjusted for age and FVC. In the receiver operating characteristic curve analysis, the 1-month change in KL-6 was also useful for predicting DP (area under the curve = 0.707; *P* < 0.012).

**Conclusions:**

Our data suggest that the relative change in KL-6 over 1 month might be useful for predicting DP in patients with IPF receiving antifibrotic therapy when baseline KL6 is high.

**Supplementary Information:**

The online version contains supplementary material available at 10.1186/s12931-022-02273-6.

## Background

Idiopathic pulmonary fibrosis (IPF) is a chronic progressive fibrosing interstitial pneumonia of unknown cause with poor prognosis. The median survival time following diagnosis, without treatment, is about 3–5 years [1–5]. Clinical trial results revealed that two antifibrotic agents—pirfenidone and nintedanib—have the capability to significantly reduce lung function decline in patients with IPF [6–8]. However, these drugs do not stop or reverse the disease progression of IPF, and treatment response varies on a case-by-case basis in previous studies [8–11]; in one study, during antifibrotic therapy, 16.5% of patients with IPF showed an absolute decline of 10% in forced vital capacity (FVC) over 1 year, whereas 83.5% of patients showed a decline of less than 10% or no decline in FVC [8]. Therefore, predicting the treatment response of antifibrotics may be useful for deciding appropriate treatment plans such as drug selection or lung transplantation. Predictive biomarkers have been evaluated for identification and assessment of disease progression (DP) in patients with IPF who were enrolled in phase 3 clinical trials of antifibrotic agents [12, 13], and baseline surfactant protein D was the only biomarker predicting the response to antifibrotics [12]. However, it has not been confirmed whether the evaluation of this marker is useful in clinical settings.

The Krebs von den Lungen-6 (KL-6) is a mucin-like glycoprotein expressed on type 2 alveolar epithelial cells, and its production increases when the lung is damaged [14–16]. We recently demonstrated that the relative changes in KL-6 over 1 week in patients with acute exacerbation of interstitial lung disease (AE-ILD) were independently associated with in-hospital mortality and that a marked change in KL-6 level (≥ 10%) is useful for differentiating patients with poor prognosis [17]. Although there have been some studies on the role of baseline or changes in KL-6 levels for predicting the DP among patients with ILD or IPF [18–20], these studies assessed outcomes regardless of antifibrotic treatment. Therefore, in this study, we aimed to determine the role of short-term changes in KL-6 in predicting DP in patients with IPF treated with antifibrotic agents.

## Methods

### Study design and population

**Fig. 1:**
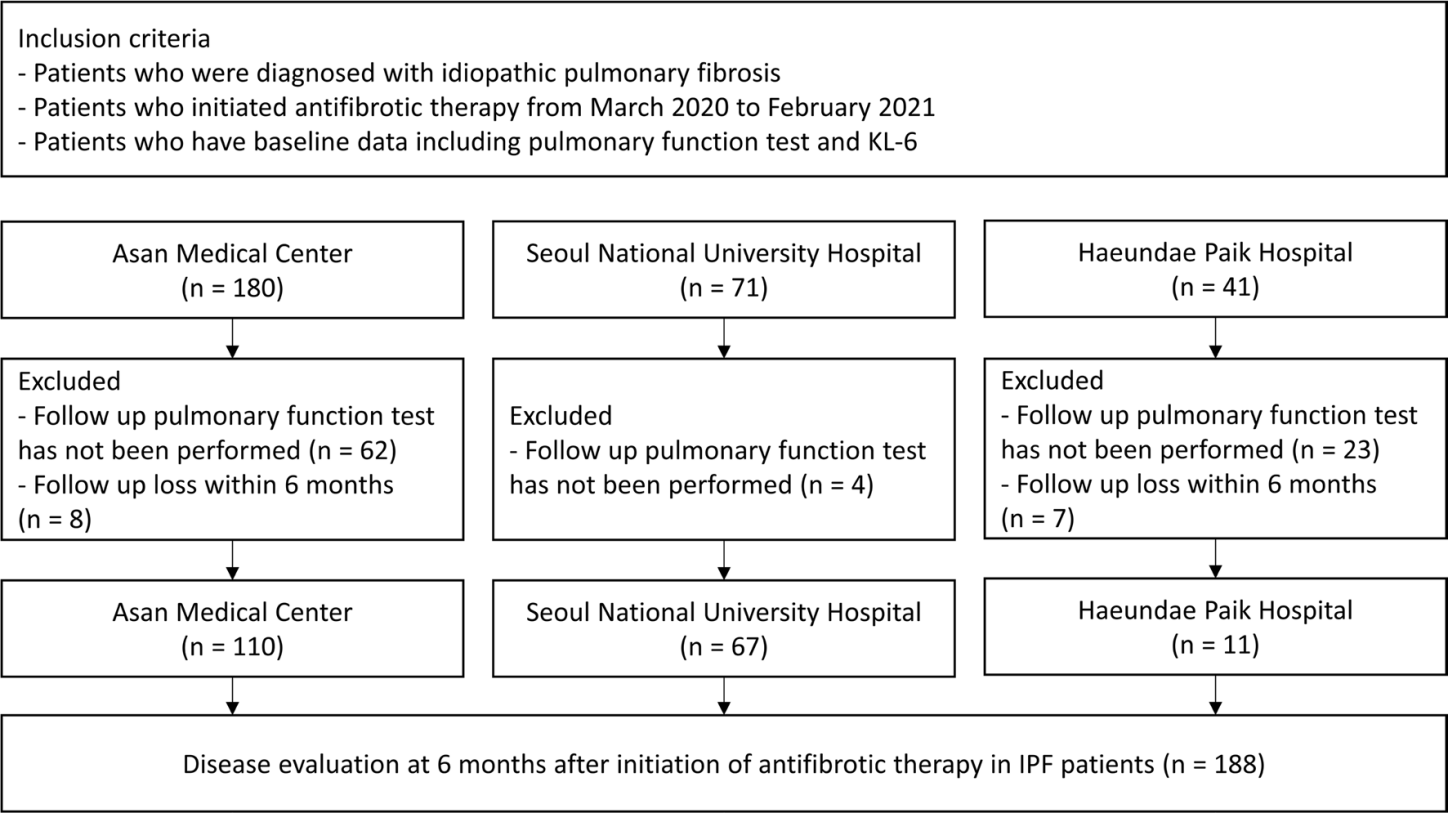
Flowchart of the study population inclusion. IPF: idiopathic pulmonary fibrosis

From March 2020 to February 2021, 292 patients with IPF who initiated antifibrotic therapy at three hospitals and had baseline data of KL-6 and lung function were screened. Of these, 104 were excluded due to loss to follow-up within 6 months or absence of serial lung function data. Finally, 188 patients were included in this study (110 at the Asan Medical Center, 67 at Seoul National University Hospital, and 11 at Inje University Haeundae Paik Hospital). Of these patients, 77 with serial KL-6 data were included in the analysis for changes in KL-6 levels (Fig. 1). IPF was diagnosed according to the 2018 American Thoracic Society (ATS)/European Respiratory Society (ERS)/Japanese Respiratory Society/Latin American Thoracic Association statement [1].

This study was approved by the Institutional Review Boards of the Asan Medical Center (approval number: 2021–0205), Seoul National University Hospital (approval number: H-2109-064-1254), and Haeundae Paik Hospital (approval number: 2021-07-007) and conducted in accordance with the ethical standards of the Declaration of Helsinki. The requirement for written informed consent was waived due to the retrospective nature of this study.

### Data collection

Clinical and survival data for all patients were retrospectively collected from medical record in each hospital. The index date was set as the date of the first prescription of antifibrotic agents. Spirometric parameters, diffusing capacity of the lung for carbon monoxide (DLco), and total lung capacity (TLC) were measured according to the ERS/ATS recommendations [21–23], and the results are presented as percentages of the normal predicted values. The results of the 6-minute walk test (6MWT), according to the recommendations of ERS/ATS, were also recorded [24].

The primary outcome of interest of this study was DP, which was defined as the relative decline in FVC ≥ 10% or DLco ≥ 15%, AE, or deaths over 6 months after antifibrotic therapy. AE was defined as an acute worsening of dyspnea, typically within 1 month, with new bilateral lung infiltration that is not fully explained by cardiac failure or fluid overload as suggested by Collard et al. [25].

### Measurement of serum KL-6

The Nanopia KL-6 assay (SEKISUI MEDICAL, Tokyo, Japan) was used to measure blood KL-6 levels. All serum samples were immediately transported to the central lab of each hospital and centrifuged after blood collection. The latex-enhanced immunoturbidimetric assay, which measures changes in absorbance by agglutination, was used to measure KL-6 concentration.

### Statistical analysis

Student’s t-test or one-way ANOVA were used for the comparison of continuous variables, with results denoted as mean ± standard deviation. The Chi-squared test was used to compare categorical variables, and values are presented as number (percentage). A logistic regression analysis was used to evaluate the risk factors for DP in patients with IPF. Variables with a *P*-value < 0.1 in the unadjusted analysis were selected and adjusted by age and FVC in the multivariable analysis. Absolute and relative changes in KL-6 values from baseline were calculated as follows: absolute change = KL-6 at follow-up − KL-6 at baseline; and relative change = (KL-6 at follow-up − KL-6 at baseline)/KL-6 at baseline × 100 (%). A receiver operating characteristic (ROC) curve analysis was used to identify the best cut-off level of changes in KL-6 in predicting DP in patients with IPF. The correlation between KL-6 and FVC changes was determined using the Pearson correlation coefficient. Subgroup analysis was also performed in patients with baseline KL-6 level ≥ 500 U/mL, which is the cut-off value for distinguishing patients with ILDs from healthy controls or those other lung diseases; this cut-off also serves as a reference value for a high level of KL-6 per the manufacturer’s recommendation [15]. A *P*-value < 0.05 was considered significant. Statistical analyses were performed using IBM SPSS version 25.0 (IBM Corp., Armonk, NY, USA).

## Results

### Baseline characteristics

The mean age of the patients was 68.9 years, and 77.7% of patients were male. The median follow-up period after the initiation of antifibrotic therapy was 7.6 (interquartile range 6.2–9.8) months. There were differences in baseline characteristics among the three hospitals, including the proportion of male patients; FVC, DLco, and TLC values; and the lowest oxygen saturation during 6MWT (Additional file 1: Table S1).

At 6 months after initiating antifibrotic agents, DP was noted in 43 patients (22.9%; number of cases of decline in FVC ≥ 10% or DLco ≥ 15%, 30; number of cases with AE, 12; number of deaths, 1). At baseline, the DP group showed older age, lower body mass index (BMI) and FVC, and shorter 6-minute walk distance (6MWD) than those in the no-DP group; however, there was no difference in baseline KL-6 levels between two groups (Table 1).

**Table 1:** Comparison of baseline characteristics between the DP and no-DP groups among patients with IPF

	Total	DP	no-DP	*P*-value
Number of patients	188	43	145	
Age, year	68.9 ± 7.7	71.2 ± 7.1	68.3 ± 7.7	0.025
Male sex	146 (77.7)	33 (76.7)	113 (77.9)	0.870
Ever-smoker	144 (76.6)	32 (74.4)	112 (77.2)	0.701
BMI, kg/m^2^	24.5 ± 3.3	23.3 ± 3.8	24.9 ± 3.1	0.011
Pulmonary function test				
FVC, % predicted	74.7 ± 17.0	69.1 ± 19.3	76.4 ± 15.9	0.013
DLco, % predicted	58.4 ± 18.5	57.0 ± 21.9	58.8 ± 17.4	0.601
TLC, % predicted	73.6 ± 13.6	70.7 ± 17.3	74.4 ± 12.3	0.196
6-minute walk test				
Distance, m	431.4 ± 109.9	377.9 ± 135.7	447.7 ± 95.6	0.005
Lowest SpO2, %	89.6 ± 6.3	88.7 ± 6.4	89.8 ± 6.2	0.309
KL-6, U/mL	1039.7 ± 823.7	1101.9 ± 955.2	1021.3 ± 783.3	0.574

Data are presented as mean ± standard deviation or number (%)

DP: disease progression; IPF: idiopathic pulmonary fibrosis; BMI: body mass index; FVC: forced vital capacity; DLco: diffusing capacity of the lung for carbon monoxide; TLC: total lung capacity; and KL-6: Krebs von den Lungen-6

When classified according to baseline KL-6 levels, patients with high baseline KL-6 level (≥ 500 U/mL, n = 144) showed younger age, lower lung function (FVC, DLco, and TLC) and poorer exercise capacity (distance and the lowest oxygen saturation during 6MWT) than patients with baseline KL-6 < 500 U/mL (Additional file 1: Table S2). Among patients with high baseline KL-6 level (≥ 500 U/mL), DP occurred in 33 patients (22.9%; number of cases of decline in FVC or DLco, 21; AE, 11; deaths, 1), and the DP group was older and had lower BMI and FVC and shorter 6MWD than the no-DP group (Additional file 1: Table S3).

### Changes in KL-6 levels

**Fig. 2:**
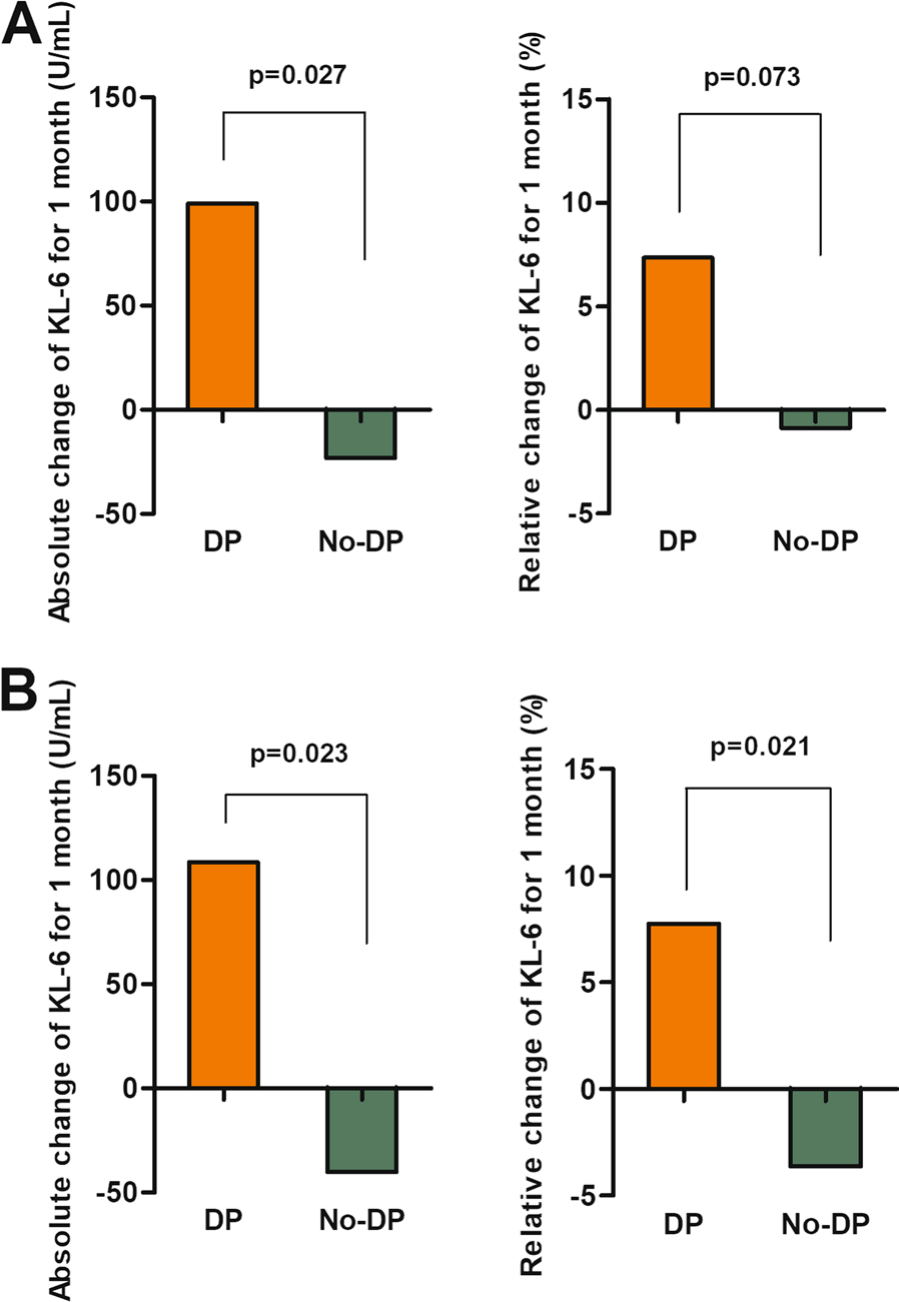
Comparison of changes in KL-6 levels according to DP in patients with IPF. **a** All patients and **b** patients with high baseline KL-6 levels (≥ 500 U/mL). KL-6: Krebs von den Lungen-6; and DP: disease progression

Among patients with IPF with serial KL-6 data (n = 77), changes in KL-6 levels over 1 month tended to be higher in the DP group ([absolute] 99.0 vs. − 23.2 U/mL, p = 0.027; [relative] 7.4 vs. − 0.9%, p = 0.073) than those in the no-DP group (Fig. 2A). Moreover, in patients with high baseline KL-6 levels (≥ 500 U/mL; n = 62), the DP group showed higher changes in KL-6 levels over 1 month ([absolute] 108.4 vs. − 40.2 U/mL, p = 0.023; [relative] 7.7 vs. − 3.6%, p = 0.021) than the no-DP group (Fig. 2B); however, there were no differences in KL-6 levels (baseline and changes over 1 month) between the two groups among patients with IPF with low baseline KL-6 levels (< 500 U/mL; n = 15).

### Prognostic factors for disease progression

In the unadjusted logistic regression analysis, lower BMI and FVC, shorter 6MWD, and higher changes in KL-6 levels (for 1 month) were risk factors for DP. In the multivariable analysis adjusted for age and FVC, lower BMI and shorter 6MWD were independent risk factors for DP; however, changes in KL-6 level over 1 month showed no significance (Table 2).

**Table 2:** Logistic regression analysis for DP in patients with IPF

Variables	Unadjusted			Multivariable (adjusted by age and FVC)		
	Odds ratio	95% CI	*P*-value	Odds ratio	95% CI	*P*-value
Age	1.057	1.007–1.109	0.026	–	–	–
Male sex	0.935	0.416–2.099	0.870	–	–	–
Ever-smoker	0.857	0.390–1.884	0.701	–	–	–
BMI, kg/m^2^	0.849	0.759–0.951	0.005	0.870	0.777–0.975	0.017
FVC, % predicted	0.973	0.952–0.995	0.015	–	–	–
DLco, % predicted	0.995	0.975–1.015	0.599	–	–	–
TLC, % predicted	0.979	0.947–1.011	0.196	–	–	–
6MWT, distance, m	0.994	0.991–0.998	0.001	0.996	0.992–1.000	0.035
6MWT, SpO2, %	0.971	0.918–1.027	0.308	–	–	–
Baseline KL-6, U/mL	1.000	1.000–1.001	0.104	–	–	–
Δ KL-6 (absolute), U/mL	1.003	1.000–1.006	0.039	1.002	0.999–1.005	0.111
Δ KL-6 (relative), %	1.029	0.997–1.063	0.077	1.028	0.993–1.064	0.120

DP: disease progression; CI: confidence interval; BMI: body mass index; FVC: forced vital capacity; DLco: diffusing capacity of the lung for carbon monoxide; TLC: total lung capacity; 6MWT: 6-minute walk test; KL-6: Krebs von den Lungen-6; and Δ: changes from baseline for 1 month

On the other hand, in patients with high baseline KL-6 level (≥ 500 U/mL), higher relative changes in KL-6 levels were independently associated with DP (odds ratio [OR] 1.043, 95% confidence interval [CI] 1.005–1.084, p = 0.028) along with lower BMI and shorter 6MWD in the multivariable analysis adjusted for age and FVC (Table 3).

**Table 3:** Logistic regression analysis for DP in IPF patients with high baseline KL-6 levels (≥ 500 U/mL)

Variables	Unadjusted			Multivariable (adjusted by age and FVC)		
	Odds ratio	95% CI	*P*-value	Odds ratio	95% CI	*P*-value
Age	1.061	1.004–1.122	0.037	–	–	–
Male sex	0.900	0.374–2.164	0.813	–	–	–
Ever-smoker	0.776	0.329–1.828	0.562	–	–	–
BMI, kg/m^2^	0.827	0.720–0.949	0.007	0.841	0.734–0.963	0.012
FVC, % predicted	0.963	0.937–0.990	0.007	–	–	–
DLco, % predicted	0.988	0.966–1.011	0.296	–	–	–
TLC, % predicted	0.971	0.934–1.009	0.130	–	–	–
6MWT, distance, m	0.993	0.990–0.997	0.001	0.995	0.991–0.999	0.019
6MWT, SpO2, %	0.975	0.918–1.036	0.421	–	–	–
Baseline KL-6	1.000	1.000–1.001	0.576	–	–	–
Δ KL-6 (absolute), U/mL	1.003	1.000–1.006	0.036	1.003	1.000–1.006	0.058
Δ KL-6 (relative), %	1.042	1.005–1.081	0.026	1.043	1.005–1.084	0.028

DP: disease progression; CI: confidence interval; BMI: body mass index; FVC: forced vital capacity; DLco: diffusing capacity of the lung for carbon monoxide; TLC: total lung capacity; 6MWT: 6-minute walk test; KL-6: Krebs von den Lungen-6; and Δ: changes from baseline for 1 month

### Clinical outcomes according to changes in KL-6 levels

**Fig. 3:**
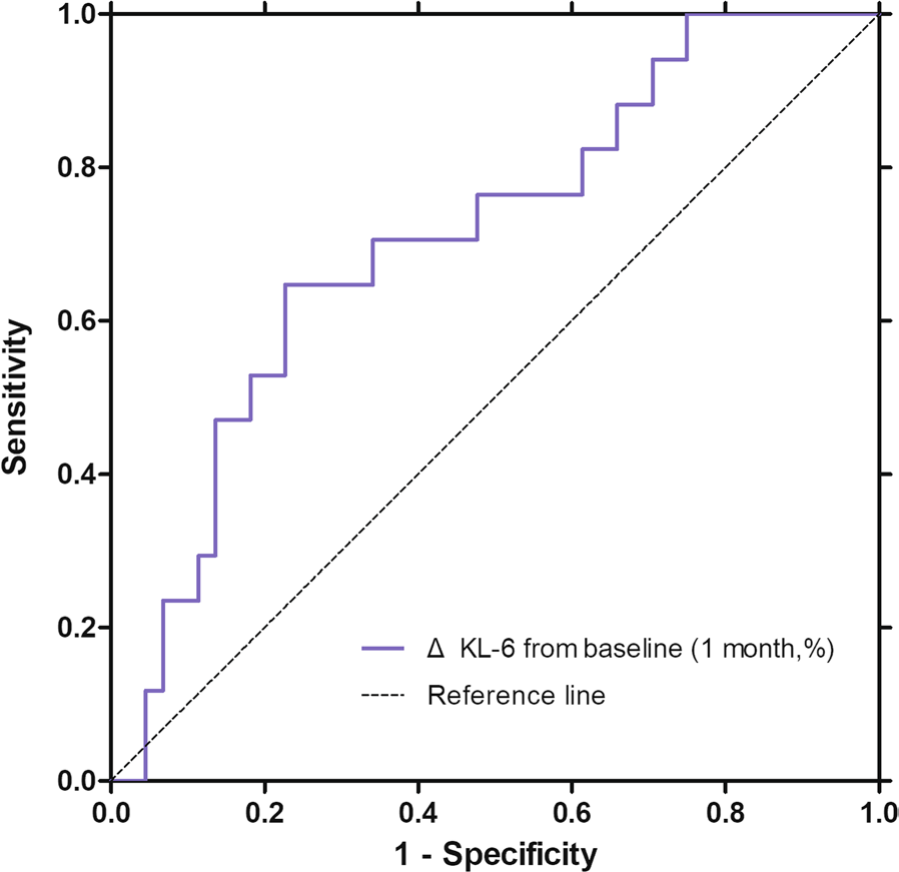
ROC curve of KL-6 changes for predicting DP in IPF patients with high baseline KL-6 levels. The area under the curve of KL-6 changes (over 1 month) for predicting DP is 0.707 (p = 0.029), and the best cut-off level is + 5% (sensitivity = 64.7%; specificity = 75.6%) in IPF patients with high baseline KL-6 levels (≥ 500 U/mL, n = 144). ROC: receiver operating characteristic; IPF: idiopathic pulmonary fibrosis; AUC: area under the curve; and KL-6: Krebs von den Lungen-6

In patients with IPF with high baseline KL-6 levels (≥ 500 U/mL, n = 144), relative changes in KL-6 levels (over 1 month) were useful for predicting DP (area under the curve = 0.707, p = 0.029) in the ROC curve analysis (Fig. 3); the best cut-off level was a 5% increase (sensitivity = 64.7%; specificity = 75.6%). Patients with marked increase in KL-6 levels (≥ 5% from baseline over 1 month) showed more frequent DP and AE and a higher decline in FVC over 6 months than those without (Table 4).

**Table 4:** Clinical outcomes according to changes in KL-6 levels in IPF patients with high baseline KL-6 levels (≥ 500 U/mL)

	Total	ΔKL-6 ≥ + 5%,	ΔKL-6 < + 5%,	*P*-value
Number of patients	62	22	40	
Disease progression	17 (27.4)	11 (50.0)	6 (15.0)	0.003
Acute exacerbation	5 (8.1)	4 (18.2)	1 (2.5)	0.030
Δ FVC, %	+ 1.0 ± 12.1	− 3.5 ± 12.9	+ 3.0 ± 10.6	0.037
Δ DLco, %	− 2.8 ± 8.5	− 4.9 ± 8.4	− 1.6 ± 8.5	0.162

KL-6: Krebs von den Lungen-6; IPF: idiopathic pulmonary fibrosis; Δ: relative changes from baseline for 1 month; FVC: forced vital capacity; and DLco: diffusing capacity of the lung for carbon monoxide

**Fig. 4:**
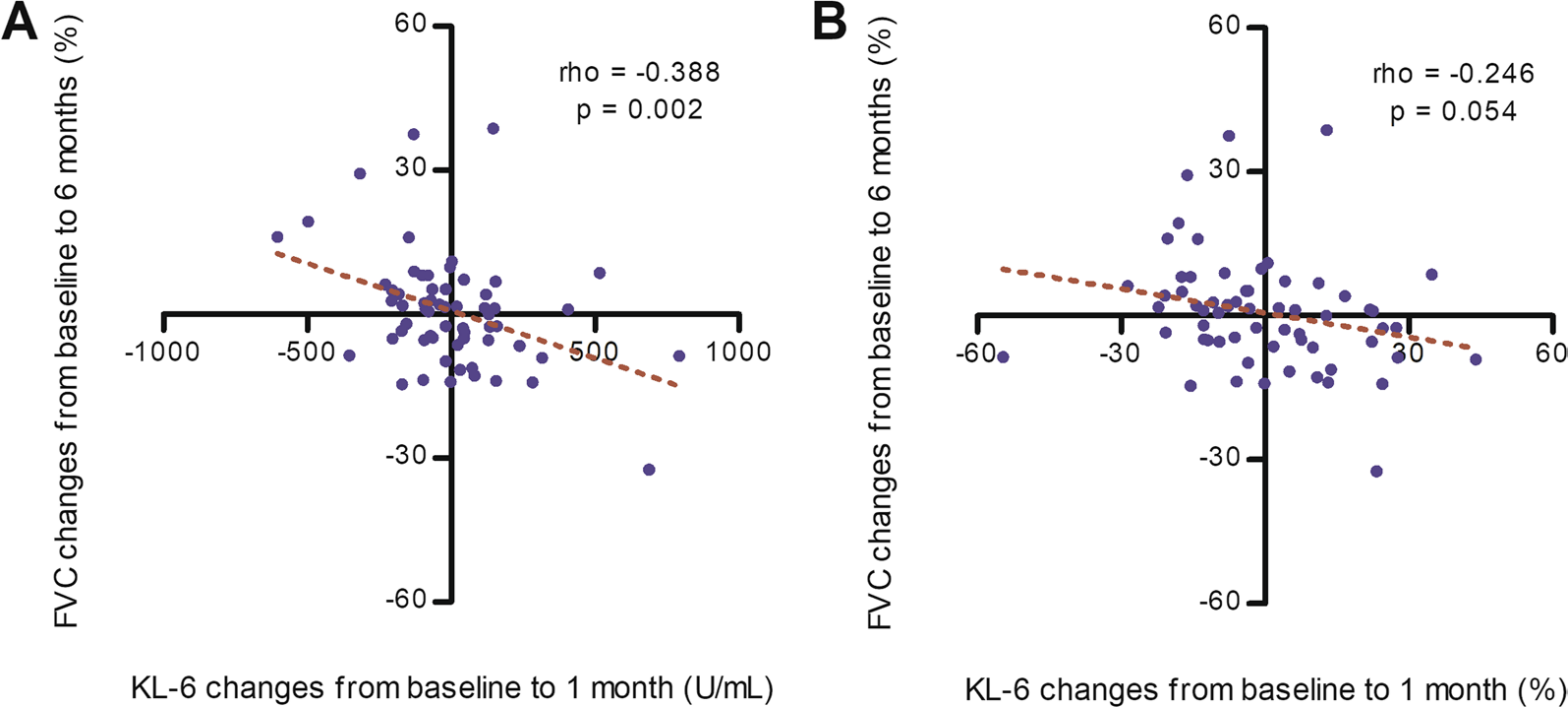
Correlation between relative FVC and KL-6 level changes in patients with high baseline KL-6 level. Correlation between relative changes in FVC (over 6 months) and **a** absolute and **b** relative change in KL-6 levels (over 1 month) in patients with high baseline KL-6 levels (≥ 500 U/mL). KL-6: Krebs von den Lungen-6; FVC: forced vital capacity

Moreover, in patients with IPF with high baseline KL-6 levels (n = 144), changes in KL-6 levels over 1 month inversely correlated with FVC relative change over 6 months ([absolute] r = − 0.388, p = 0.002; [relative] r = − 0.246, p = 0.054) (Fig. 4).

## Discussion

In our study, the short-term changes in KL-6 levels were associated with DP in patients with IPF receiving antifibrotic agents. Particularly in patients with high baseline KL-6 levels (≥ 500 U/mL), the relative changes in KL-6 levels over 1 month were independently associated with DP, and those with a remarkable increase in KL-6 ( ≥ + 5%) had poor outcomes. Moreover, in this group, the short-term changes in KL-6 levels correlated with FVC changes over 6 months.

Few recent studies have studied biomarkers for predicting treatment response in patients with IPF receiving antifibrotic agents [12, 13]. Ikeda et al., in the post-hoc analysis of the phase 3 trial of pirfenidone in Japan, showed that baseline serum surfactant protein D level (OR, 1.003; 95% CI 1.000–1.006, p = 0.028) predicted DP (≥ 10% relative decline in FVC from baseline and/or death over 1 year after treatment) in the pirfenidone group (n = 163) in the multivariate logistic analysis adjusted for smoking, BMI, FVC and alveolar–arterial oxygen difference [12]. Neighbors et al., in a post-hoc assessment of the CAPACITY (n = 184) and ASCEND (n = 229) trials, also investigated the association between the levels of various biomarkers (CCL13, CCL17, CCL18, CXCL13, CXCL14, COMP, interleukin 13, MMP3, MMP7, osteopontin, periostin, and YKL40) and absolute decline in FVC (%) over 12 months in patients with IPF treated with pirfenidone; however, they were unable to find biomarkers associated with the treatment response to antifibrotic agents [13]. These studies did not reflect the results in real-world patients with IPF with varying severity because they only included IPF patients with mild-to-moderate severity who were enrolled in those clinical trials. Our results showed that changes in KL-6 levels were associated with treatment response to antifibrotic agents in real-world patients with IPF.

Several studies have shown an association between changes in KL-6 levels and the clinical course of patients with IPF [18, 20]. Sokai et al. showed that the absolute changes in serum KL-6 level over 6 months were significantly correlated with the absolute changes in FVC and DLco over 6 months (r = − 0.38, p < 0.01; r = − 0.33, p < 0.01, respectively) among 75 patents with IPF, 14.7% of whom received pirfenidone [20]. Jiang et al., among 20 patients with IPF (no data on the proportion of patients with antifibrotic agents), also reported that the absolute changes in KL-6 levels within 1–6 months before disease evaluation were prognostic factors for DP (OR, 3.611; 95% CI 1.05–6.22; p < 0.01), which was defined as ≥ 10% absolute decline in FVC or ≥ 15% absolute decline in DLco from baseline over 12 months in the multivariate logistic analysis adjusted for by age, lung function, and disease extent as assessed using high-resolution computed tomography [18]. Wakmatsu et al. reported that patients with increased KL-6 levels during follow-up (≥ 6 months) had a higher annual decline in FVC than that in those without increased levels of KL-6 (− 201 vs. − 50.7 mL/year, p < 0.001) among 66 patients who received pirfenidone [26]. These results suggest that the changes in KL-6 might be associated with the clinical course even in patients receiving antifibrotic therapy. Moreover, compared to other variables including BMI and 6MWD, KL-6 could better predict treatment response by reflecting the pathophysiology of disease progression characterized by alveolar epithelial cell injury [15, 27].

Interestingly, in our study, relative changes in KL-6 levels over 1 month predicted DP, but only in patients with high baseline KL-6 levels. Although the reason behind the discrepancy in results depending on baseline KL-6 levels is unclear, a previous study reported that baseline KL-6 levels are not elevated in some patients with IPF with a low fibrosis score on high-resolution computed tomography or high FVC values [19, 28]. Thus, we hypothesized that KL-6 could not reflect disease activity well in patients with IPF with low baseline KL-6 levels, which might be associated with mild disease severity.

Meanwhile, there have been studies that reported the association between baseline KL-6 levels and DP in patients with ILD [19, 29]. Ko et al., among 199 patients with ILD (IPF = 43; 19.6% received antifibrotic agent), showed that a high baseline KL-6 level (≥ 800 U/mL) was a poor prognostic factor for DP (≥ 10% relative decline in FVC from baseline or AE over 1 year after diagnosis) (hazard ratio [HR], 2.689; 95% CI 1.445–5.004; p = 0.002) in the multivariable Cox analysis adjusted for by age, FVC, and lowest oxygen saturation during 6MWT [19]. Stock et al., among 189 patients with systemic sclerosis-associated ILD, also reported that the patients with high baseline KL-6 levels (> 1472 U/mL) showed shorter time to ≥ 15% decline in DLco (p = 0.003) [29]. However, in our study, baseline KL-6 was not associated with DP. The use of antifibrotics in patients with IPF could slow DP [30], which might weaken the prognostic value of baseline KL-6 levels.

Baseline BMI and 6MWD were prognostic factors for the DP of IPF in our study. The results of previous studies are consistent with those reported herein [12, 31]. Ikeda et al., among 163 IPF patients receiving pirfenidone, showed that lower baseline BMI and FVC were poor prognostic factors ([FVC] OR, 0.93; 95% CI 0.916–0.971; p < 0.0001; [BMI] OR, 0.849; 95% CI 0.723–0.998; p = 0.0469) for DP (≥ 10% relative decline in FVC from baseline and/or death over 1 year after antifibrotic therapy) in the multivariate logistic analysis adjusted for smoking, alveolar-arterial oxygen gradient, and surfactant protein D [12]. Another study, in 748 patients with IPF from the INSPIRE trial, also showed that shorter baseline 6MWD (< 250 m) was a poor prognostic factor for 1-year mortality (HR, 2.12; 95% CI 1.15–3.92; p = 0.02) in the multivariate Cox analysis adjusted for age and FVC [31].

This study has some limitations. First, this study had a retrospective design and was conducted only in Korean patients, which may limit the generalizability of our findings. However, the demographic features and lung function of patients included in this study (FVC, 74.7%; DLco, 58.4%) were comparable with those reported in other studies (FVC, 74–78%; DLco, 47–56%) [12, 13, 19]. Second, the effect of antifibrotics on KL-6 levels was unclear because there was no comparison with patients with IPF who did not receive pirfenidone. However, it is difficult to extract data on untreated controls in this era when antifibrotic treatment is the standard of care for patients with IPF [32]. Nevertheless, unlike other studies that showed the role of KL-6 in predicting the prognosis of IPF regardless of antifibrotic treatment, we could infer that the role of KL-6 change is to predict treatment response by evaluating DP in patients with IPF who received antifibrotic agents. Third, the median follow-up period was short. However, all patients were followed up at least 6 months after treatment to determine significant prognostic factors for DP. Finally, there were differences in baseline characteristics, including age, sex, and lung function, among patients according to centers or baseline KL-6 levels. However, in the multivariable analysis, variables were adjusted by age and FVC.

## Conclusions

In conclusion, our data suggest that, in patients with a high baseline KL-6 level (≥ 500 U/mL), the short-term changes in KL-6 levels might be useful in predicting DP during antifibrotic treatment and reflect longer-term changes in lung function.

## Supplementary Information

**Additional file  1: Table S1.** Comparison of the baseline characteristics among patients with IPF according to participating centers. **Table S2.** Comparison of the baseline characteristics among patients with IPF according to baseline KL-6 levels. **Table S3.** Comparison of the baseline characteristics between the DP and no-DP groups among patients with IPF with high baseline KL-6 levels (≥ 500 U/mL).

## Data Availability

The datasets used and/or analyzed in the current study are available from the corresponding author upon reasonable request.

## Abbreviations

6MWD: 6-Minute walk distance
6MWT: 6-Minute walk test
AE: Acute exacerbation
ATS: American Thoracic Society
BMI: Body mass index
DLco: Diffusing capacity of the lung for carbon monoxide
DP: Disease progression
ERS: European Respiratory Society
FVC: Forced vital capacity
HR: Hazard ratio
ILD: Interstitial lung disease
IPF: Idiopathic pulmonary fibrosis
KL-6: Krebs von den Lungen-6
OR: Odds ratio
ROC: Receiver operating characteristic
TLC: Total lung capacity
