# Supplementary material for: Blood Krebs von den Lungen-6 levels predict treatment response to antifibrotic therapy in patients with idiopathic pulmonary fibrosis
Source: Respir Res. 2022 Dec 9;23:334. doi: 10.1186/s12931-022-02273-6 (PMC9733030; doi:10.1186/s12931-022-02273-6)
Supplement: Supplementary file 1 — Additional file 1: Table S1. Comparison of the baseline characteristics among patients with IPF according to participating centers. Table S2. Comparison of the baseline characteristics among patients with IPF according to baseline KL-6 levels. Table S3. Comparison of the baseline characteristics between the DP and no-DP groups among patients with IPF with high baseline KL-6 levels (≥ 500 U/mL). [file 12931_2022_2273_MOESM1_ESM.docx]

**ADDITIONAL FILE**

**Blood Krebs von den Lungen-6 levels predict treatment response to antifibrotic therapy in patients with idiopathic pulmonary fibrosis**

Myeong Geun Choi^1, 2^, Sun Mi Choi^3^, Jae Ha Lee^4^, Joong-Yub Kim^3^, Jin Woo Song^1*^

^1^ Department of Pulmonary and Critical Care Medicine, Asan Medical Center, University of Ulsan College of Medicine, Seoul, South Korea

^2^ Division of Pulmonary and Critical Care Medicine, Department of Internal Medicine, Mokdong Hospital, College of Medicine, Ewha Womans University, Seoul, South Korea

^3^ Division of Pulmonary and Critical Care Medicine, Department of Internal Medicine, Seoul National University Hospital, Seoul National University College of Medicine, Seoul, South Korea

^4^ Division of Pulmonary and Critical Care Medicine, Department of Internal Medicine, Inje University Haeundae Paik Hospital, Inje University College of Medicine, Busan, South Korea

Table S1. Comparison of baseline characteristics among patients with IPF according to participating centers.

|  | Total | AMC | SNUH | IUHPH | P-value |
| --- | --- | --- | --- | --- | --- |
| Number of patients | 188 | 110 | 67 | 11 |  |
| Age, year | 68.9 ± 7.7 | 67.8 ± 8.2 | 70.3 ± 6.7 | 71.6 ± 6.6 | 0.055 |
| Male sex | 146 (77.7) | 84 (76.4) | 57 (85.1) | 5 (45.5) | 0.012 |
| Ever-smoker | 144 (76.6) | 84 (76.4) | 54 (80.6) | 6 (54.5) | 0.103 |
| BMI, kg/m^2^ | 24.5 ± 3.3 | 24.5 ± 3.5 | 24.7 ± 3.1 | 23.7 ± 3.4 | 0.663 |
| Pulmonary function test |  |  |  |  |  |
| FVC, % predicted | 74.7 ± 17.0 | 71.4 ± 15.2 | 79.8 ± 18.8 | 77.5 ± 15.3 | 0.005 |
| DLco, % predicted | 58.4 ± 18.5 | 55.5 ± 17.5 | 65.8 ± 19.7 | 55.5 ± 14.4 | 0.005 |
| TLC, % predicted | 73.6 ± 13.6 | 71.3 ± 12.7 | 82.1 ± 14.1 | - | 0.001 |
| 6-minute walk test |  |  |  |  |  |
| Distance, m | 431.4 ± 109.9 | 417.1 ± 111.3 | 451.7 ± 103.7 | 467.4 ± 113.6 | 0.101 |
| Lowest SpO2, % | 89.6 ± 6.3 | 90.6 ± 5.7 | 87.7 ± 7.0 | 89.6 ± 4.9 | 0.025 |
| KL-6, U/mL | 1039.7 ± 823.7 | 993.3 ± 735.0 | 1125.8 ± 886.5 | 979.3 ± 1239.9 | 0.568 |

Data are presented as mean ± standard deviation, median (interquartile range), or number (%).

IPF, idiopathic pulmonary fibrosis; AMC, Asan Medical Center; SNUH, Seoul National University Hospital; IUHPH, Inje University Haeundae Paik Hospital; KL-6, Krebs von den Lungen-6; BMI, body mass index; FVC, forced vital capacity; DLco, diffusing capacity of the lung for carbon monoxide; and TLC, total lung capacity.

Table S2. Comparison of baseline characteristics of patients with IPF according to baseline KL-6 levels

|  | Total | Baseline KL-6  ≥500 U/mL | Baseline KL-6  <500 U/mL | P-value |
| --- | --- | --- | --- | --- |
| Number of patients | 188 | 144 | 44 |  |
| Age, year | 68.9 ± 7.7 | 68.8 ± 7.8 | 71.6 ± 6.6 | 0.008 |
| Male sex | 146 (77.7) | 107 (74.3) | 39 (88.6) | 0.061 |
| Ever-smoker | 144 (76.6) | 106 (73.6) | 38 (86.4) | 0.104 |
| BMI, kg/m^2^ | 24.5 ± 3.3 | 24.6 ± 3.2 | 24.2 ± 9.7 | 0.459 |
| Pulmonary function test |  |  |  |  |
| FVC, % predicted | 74.7 ± 17.0 | 72.7 ± 16.6 | 81.3 ± 16.6 | 0.003 |
| DLco, % predicted | 58.4 ± 18.5 | 55.8 ± 19.1 | 66.8 ± 13.6 | < 0.001 |
| TLC, % predicted | 73.6 ± 13.6 | 71.6 ± 13.4 | 80.1 ± 12.4 | 0.003 |
| 6-minute walk test |  |  |  |  |
| Distance, m | 431.4 ± 109.9 | 417.7 ± 112.6 | 479.7 ± 85.0 | 0.003 |
| Lowest SpO2, % | 89.6 ± 6.3 | 88.6 ± 6.6 | 93.0 ± 3.2 | < 0.001 |
| KL-6, U/mL | 1039.7 ± 823.7 | 1239.6 ± 844.8 | 385.6 ± 76.8 | < 0.001 |

Data are presented as mean ± standard deviation, median (interquartile range), or number (%).

IPF, idiopathic pulmonary fibrosis; KL-6, Krebs von den Lungen-6; BMI, body mass index; FVC, forced vital capacity; DLco, diffusing capacity of the lung for carbon monoxide; and TLC, total lung capacity.

Table S3. Comparison of baseline characteristics between the DP and no-DP groups among patients with IPF with high baseline KL-6 levels (≥ 500 U/mL)

|  | Total | DP | no-DP | *P*-value |
| --- | --- | --- | --- | --- |
| Number of patients | 144 | 33 | 111 |  |
| Age, year | 68.8 ± 7.8 | 70.4 ± 7.4 | 67.4 ± 7.8 | 0.034 |
| Male sex | 107 (74.3) | 24 (72.7) | 83 (74.8) | 0.823 |
| Ever-smoker | 106 (73.6) | 23 (70.0) | 83 (74.8) | 0.496 |
| BMI, kg/m^2^ | 24.6 ± 3.2 | 23.3 ± 3.5 | 25.1 ± 3.1 | 0.006 |
| Pulmonary function test |  |  |  |  |
| FVC, % predicted | 72.7 ± 16.6 | 65.8 ± 18.0 | 74.8 ± 15.7 | 0.006 |
| DLco, % predicted | 55.8 ± 19.1 | 52.6 ± 21.8 | 56.8 ± 18.1 | 0.298 |
| TLC, % predicted | 71.6 ± 13.4 | 68.0 ± 14.8 | 72.8 ± 12.8 | 0.128 |
| 6-minute walk test |  |  |  |  |
| Distance, m | 417.7 ± 112.6 | 352.8 ± 131.3 | 438.7 ± 97.7 | 0.002 |
| Lowest SpO2, % | 88.6 ± 6.6 | 87.8 ± 6.6 | 88.9 ± 6.6 | 0.424 |
| KL-6, U/mL | 1239.6 ± 844.8 | 1311.8 ± 1000.5 | 1218.1 ± 796.6 | 0.578 |

Data are presented as mean ± standard deviation or number (%).

DP, disease progression; IPF, idiopathic pulmonary fibrosis; BMI, body mass index; FVC, forced vital capacity; DLco, diffusing capacity of the lung for carbon monoxide; TLC, total lung capacity; and KL-6, Krebs von den Lungen-6.
